# Urine peptidome analysis in cardiorenal syndrome reflects molecular processes

**DOI:** 10.1038/s41598-021-95695-z

**Published:** 2021-08-10

**Authors:** Eleni Petra, Tianlin He, Vasiliki Lygirou, Agnieszka Latosinska, Harald Mischak, Antonia Vlahou, Joachim Jankowski

**Affiliations:** 1 Institute for Molecular Cardiovascular Research, RWTH Aachen University Hospital, Pauwelsstraße 30, 52074 Aachen, Germany; 2 Biotechnology Division, Biomedical Research Foundation of the Academy of Athens, Athens, Greece; 3 Mosaiques Diagnostics GmbH, Hannover, Germany; 4 Experimental Vascular Pathology, Cardiovascular Research Institute Maastricht (CARIM), University of Maastricht, Maastricht, The Netherlands

**Keywords:** Biomarkers, Cardiology, Nephrology, Proteomic analysis

## Abstract

The cardiorenal syndrome (CRS) is defined as the confluence of heart-kidney dysfunction. This study investigates the molecular differences at the level of the urinary peptidome between CRS patients and controls and their association to disease pathophysiology. The urinary peptidome of CRS patients (n = 353) was matched for age and sex with controls (n = 356) at a 1:1 ratio. Changes in the CRS peptidome versus controls were identified after applying the Mann–Whitney test, followed by correction for multiple testing. Proteasix tool was applied to investigate predicted proteases involved in CRS-associated peptide generation. Overall, 559 differentially excreted urinary peptides were associated with CRS patients. Of these, 193 peptides were specifically found in CRS when comparing with heart failure and chronic kidney disease urinary peptide profiles. Proteasix predicted 18 proteases involved in > 1% of proteolytic cleavage events including multiple forms of MMPs, proprotein convertases, cathepsins and kallikrein 4. Forty-four percent of the cleavage events were produced by 3 proteases including MMP13, MMP9 and MMP2. Pathway enrichment analysis supported that ECM-related pathways, fibrosis and inflammation were represented. Collectively, our study describes the changes in urinary peptides of CRS patients and potential proteases involved in their generation, laying the basis for further validation.

## Introduction

The cardiorenal syndrome (CRS) is a complex pathological disorder, which reflects the interplay between heart and chronic kidney diseases^1^. Epidemiologic data reveal that about 45–63% of chronic heart failure (HF) patients developed chronic kidney disease (CKD)^2^. CRS is classified into five subtypes, referring to primary organ dysfunction, each with different underlying pathological mechanisms^3^. The term "CRS" has been applied to the relation of these two diseases, but the definition and classification have not been clearly established on a molecular pathophysiologic level until now^4^. However, multi-factorial mechanisms including hemodynamic changes, fibrosis, vascular calcification, neurohormonal activity, immunologic imbalance, inflammation, apoptosis, endothelial injury, thrombosis, and oxidative stress have been proposed to explain the general pathophysiology of CRS^5^.

In parallel to the efforts towards understanding disease pathophysiology, a number of biomarkers for heart and kidney disease have been studied in the context of CRS. These include changes in the levels of heart-specific biomarkers such as cardiac troponin T (cTnT) and B-type natriuretic peptide (BNP)^6,7^, but also kidney-specific biomarkers including albumin (ALB), kidney injury molecule-1 (KIM-1)^8^, liver-type fatty acid binding protein (L-FABP)^9^, neutrophil gelatinase-associated lipocalin (NGAL)^10^, β-2 microglobulin (B2M)^11^, n-acetyl-beta-d-glucosaminidase (NAG)^12^ and cystatin C^13^, (reviewed in^14^).

Urinary peptidomics focuses on the analysis of naturally occurring peptides and small proteins in urine. Application of the approach enabled identification of biomarkers associated with kidney and heart diseases including, among others, CKD^15^, kidney fibrosis^16^, diabetic kidney disease (DKD)^17^, acute kidney injury (AKI)^18^ as well as HF^19^, asymptomatic LV diastolic dysfunction (LVDD)^20^, and ischemic and dilated cardiomyopathy^21^; with a biomarker panel for CKD also having reached the level of clinical implementation for patient stratification in large clinical trials^22^.

Due to the lack of specific CRS biomarkers on a molecular level, we designed the current study to identify molecular differences at the level of the urinary peptidome between CRS and matched controls, prompted by the availability of high resolution-large sized datasets. In addition, we targeted to investigate if the observed urinary changes reflect molecular processes associated with CRS and its underlying pathologies.

## Results

### Cohort characteristics

The urine peptidomics datasets were screened for the availability of clinical information on both kidney and heart failure. Based on these criteria, 3463 urinary peptidomics datasets were retained for investigation. Matching resulted in the inclusion of a total of 709 datasets (CRS; n = 353, controls; n = 356) in the study, which comprised the main groups under comparison (CRS versus the respective matched controls). The CRS patients had an estimated glomerular filtration rate (eGFR) mean value of 43.1 ml/min/1.73 m^2^ whereas controls had 79.2 ml/min/1.73 m^2^. A number of the clinicopathological characteristics including the NT-proBNP levels, the serum creatinine levels, the systolic blood pressure, the diastolic blood pressure were statistically significant different between the two groups. The characteristics of the matched cases-controls are presented in Table 1.

**Table 1:** Demographics and clinicopathological features characterizing the CRS cohort under investigation.

	Control	CRS
Number	356	353
Sex, male (%)	67	68
Age (years)	70 ± 7	72 ± 8
BMI	26.75 ± 4.00	29.19 ± 5.29*
Smoking status (yes, %)	9.5	5.3*
eGFR (CKD-EPI) (ml/min/1.73 m^2^)	79.2 ± 18.8	43.1 ± 11.5*
CKD Stage 5 (%)	N/A	1
CKD Stage 4 (%)	N/A	14
CKD Stage 3 (%)	N/A	85
NT-proBNP (pg/mL)	195.16 ± 195.49	1587.48 ± 2111.07*
Serum creatinine (μmol/L)	79.20 ± 12.81	138.04 ± 47.15*
Ejection fraction	N/A	40.10 ± 11.92
Systolic BP (mmHg)	141.66 ± 18.13	126.91 ± 24.57*
Diastolic BP (mmHg)	79.75 ± 8.99	67.44 ± 13.16*
Diagnosis (%) HF/HFrEF/HFpEF/HFmrEF	N/A	15/60/20/5

Differences between controls and CRS have been evaluated by the Mann–Whitney U test (for continuous variables) or Chi-Square test (for categorical variables) and are marked with * when P < 0.05. Data are presented as mean ± standard deviation (s.d.) or number (%).

BMI: body mass index; eGFR: estimated glomerular filtration rate; CKD-EPI: Chronic Kidney Disease Epidemiology Collaboration; CKD Stage 5: 0–15 ml/min/1.73 m^2^; CKD Stage 4: 15–29 ml/min/1.73 m^2^; CKD Stage 3: 30–60 ml/min/1.73 m^2^; NT-proBNP: N-terminal pro b-type natriuretic peptide; BP: blood pressure; HF: heart failure; HFrEF: heart failure with reduced ejection fraction; HFpEF: heart failure with preserved ejection fraction; HFmrEF: heart failure with mid-range ejection fraction; N/A: not applicable.

### Urine peptidome analysis

**Figure 1:**
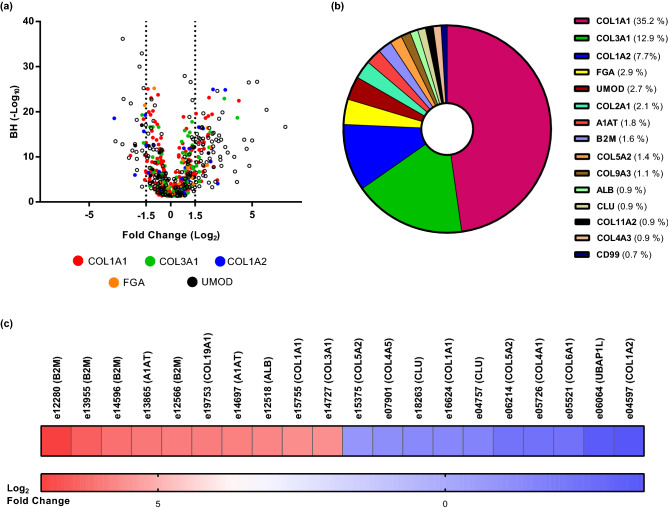
Urinary peptides in CRS. (**a**) Volcano plot showing the fold change (log_2_ CRS/Control) plotted against the BH P-value (− log_10_) of the 559 differentially excreted peptides between CRS and controls. The top five most representative protein precursors including COL1A1, COL3A1, COL1A2, FGA and UMOD are highlighted in different colors. (**b**) Pie chart of the top 15 most frequently observed protein precursors, associated with at least 400 differentially excreted CRS-associated peptides. The percentage of the differentially excreted peptides per protein precursor is presented. 55.9% of CRS-associated peptides originated from fibril-forming collagens and were fragments of COL1A1, COL1A2, and COL3A1. (**c**) Heatmap of the log_2_ transformed fold change of the top 10 most increased and decreased peptides in the CRS cohort. In each case, the peptide codes are provided in Supplementary Table S1.

A total of 3184 peptides were detected and considered when comparing CRS patients with controls. After applying the Benjamini-Hochberg (BH) adjustment, 1480 differentially excreted peptides (BH, P < 0.05) were identified when comparing cases with controls. To shortlist the more representative features, a 30% frequency threshold, frequently used in such analyses^23,24^ was applied, which resulted in a final list of 559 differentially excreted peptides (see Fig. 1a and Supplementary Table S1). These peptides derived from 110 unique protein precursors, which based on functional annotation, reflected largely extracellular matrix (ECM) changes, cell-ECM interactions, collagen formation/degradation, metabolic and inflammatory processes. The top 15 most frequently observed protein precursors, associated with at least 400 differentially excreted peptides are presented in Fig. 1b. Of these 559 differentially excreted peptides, 313 (55.9%) originated from fibril-forming collagens and were fragments of collagen type I alpha 1 chain (COL1A1), collagen type I alpha 2 chain (COL1A2), and collagen type III alpha 1 chain (COL3A1). Together the collagens accounted for 72% of the quantified peptidome. The 20 most significant differentially excreted peptides included fragments of additional collagen types (COL4A1, COL4A3, COL9A3, COL5A1, COL5A2, COL5A3 and COL19A1) and other peptides originating from plasma proteins (such as apolipoprotein A1 (APOA1) and B2M) (see Table 2). Among the peptides showing the most prominent increase in abundance in patients versus controls were fragments of B2M, albumin, and alpha-1-antitrypsin (A1AT), whereas fragments of collagen types (IV, V and VI), clusterin (CLU) and ubiquitin-associated protein 1-like (UBAP1L) were the peptides with the most decreased abundance levels in CRS (see Fig. 1c).

**Table 2:** List of the 20 top most significant differentially excreted CRS peptides.

Sequence	Protein symbol	Start AA	Stop AA	p-value (BH)	Fold change
GPpGFTGppGPPGPPGP	COL4A1	197	213	6.99E−37	*0.13*
GppGFTGPpGpPGPPGP	COL4A1	197	213	1.14E−33	*0.29*
mPGFKGpTGYKGEQGEVGKDGEKGDpGpPG	COL9A3	151	180	9.57E−31	*0.27*
PGMPGADGpPGHPGKEGppGEKGGQGpPG	COL5A1	753	781	1.24E−28	**1.81**
LLKNGERIEKVEHSDLSFSKDWS	B2M	59	81	2.21E−27	**39.40**
cDDYRLcE	MGP	73	90	2.66E−27	**2.33**
GPEGPSGKpGINGKDGIPGAQGImGKpGDRGpKGERGDQGIP	COL19A1	917	958	3.26E−27	**27.61**
GpKGDpGIpGLDRSGFpGETGSPGIPGHQ	COL4A3	836	864	5.66E−26	*0.32*
DEAGSEADHEGTHSTK	FGA	605	620	6.70E−26	*0.50*
DDGEAGKpGRpG	COL1A1	231	242	8.94E−26	*0.38*
LkGQpGApGVkGEpGApGENGTpGQTGARG	COL1A2	188	217	1.12E−25	**6.06**
GpAGPpGKAGEDGHPGKpGRpGERG	COL1A2	133	157	1.42E−25	**10.23**
VDVLKDSGRDYVSQFEGSALGKQLN	APOA1	43	67	2.32E−25	**7.26**
ApGDRGEpGPpGPAG	COL1A1	798	812	1.84E−24	*0.58*
SpGRDGSpGAKGDRGETGP	COL1A1	1023	1041	7.20E−24	**5.09**
AEGSpGRDGSpGAKGDRGETGPA	COL1A1	1020	1042	9.09E−24	*0.44*
GLAGTAGEpGRDGNPGSDGLPGRDGSpGGKGDRGENGSpGAPGAPGHPGPPGp	COL3A1	1002	1054	1.20E−23	**9.81**
GSPGTSGppGSAGpPGSpG	COL5A2	435	453	1.53E−23	*0.14*
GPpGPpGFpGDPGPPG	COL5A3	407	422	1.85E−23	*0.44*
cDDYRLc	MGP	73	79	2.75E−23	**2.54**

Peptides increased in CRS are labelled in bold, decreased in italics.

AA: amino acid; BH: Benjamin–Hochberg.

### Urinary peptide differences in CRS, CKD and HF

We further investigated if common peptides were identified between patients with HF and CRS as well as between CKD and CRS. We compared the 559 peptides of our study with the 218 and 577 differentially excreted peptides which were associated with CKD and HF, respectively, and described in previous studies^15,19^. Of these 218 CKD-associated peptides, 31 (14.2%) were commonly identified in our study as well. These common 31 peptides were sequenced fragments of albumin, B2M, A1AT, APOA1, alpha-1B-glycoprotein (A1BG), alpha-2-HS-glycoprotein (AHSG), sodium/potassium–transporting ATPase subunit γ (FXYD2), osteopontin (SPP1), collagen types I and III and other proteins (see Supplementary Table S2). In addition, 341 out of 577 (59%) of the HF-specific peptides were commonly identified in CRS patients (see Supplementary Table S3). These shared peptides showed the same directionality of difference and originated mostly from collagen types (I, II, III, IV, V, IX, XI), B2M, A1AT and uromodulin (UMOD).

Overall, 193 of 559 (34.5%) CRS-associated peptides were not among those identified in the HF and CKD studies (see Supplementary Table S4). Most of these 193 peptides, which were found in the CRS cohort only, were collagen fragments (n = 134, 69.4%), with collagen types I, II, III, IV and V represented by the largest number of collagen fragments (n = 117, 60.6%). The top most represented protein precursors were collagen types I (40.9%) and III (9.3%), UMOD (4.1%) fibrinogen alpha chain (FGA, 2.6%), COL5A2 (2.1%), CD99 (1.6%), CLU (1.6%), FXYD2 (1.6%) and polymeric immunoglobulin receptor (PIGR, 1.6%).

On a protein level, we further investigated if any of the protein precursors represented by 193 CRS-associated peptides was not identified in the CKD and HF urinary profiles. Totally, 30 protein precursors were detected only in CRS cohort but not in HF and CKD. These protein precursors included several collagen types (COL4A2, COL4A4, COL6A5, COL7A1, COL8A1 and COL13A1), PIGR, secreted and transmembrane protein 1 (SECTM1), ankyrin repeat domain-containing protein 17 (ANR17), ubiquitin-like protein ATG12 (ATG12), zinc-alpha-2-glycoprotein (AZGP1) and monocyte differentiation antigen CD14 (CD14) (and other proteins listed in Supplementary Table S5).

### Protease prediction analysis

**Figure 2:**
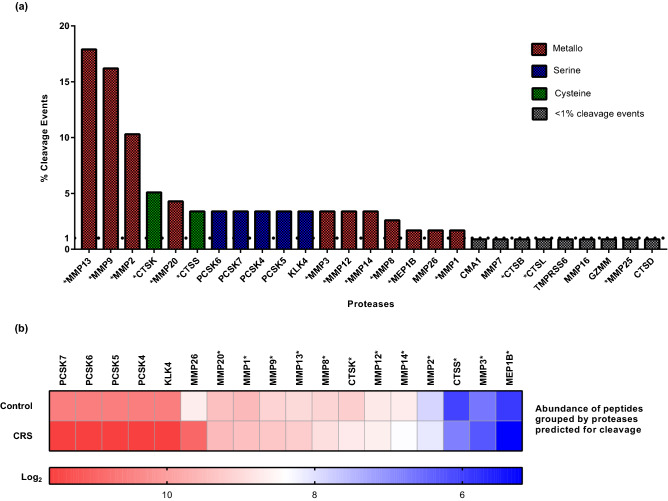
In silico predicted proteases. (**a**) The 27 predicted proteases are presented. Of these, 18 were linked to ≥ 1% of cleavage events and are highlighted in red, blue and green whereas 9 had < 1% of cleavage events (highlighted in grey). The 15 predicted proteases which were involved in the generation of the 193 CRS-specific peptides are marked with an asterisk. The catalytic mechanism of the shortlisted proteases is also depicted. (**b**) Heat map of the log_2_ transformed abundance of the differentially excreted peptides predicted as substrates of 18 shortlisted proteases by the Proteasix. The predicted proteases which were associated with the 193 CRS-specific peptides are marked with an asterisk.

Proteases possibly implicated in the endogenous cleavage of the differentially excreted peptides were predicted by Proteasix tool (http://www.proteasix.org). The in silico analysis revealed that 117 of the 559 differentially excreted peptides were known substrates of 27 reported in the literature proteases. Of these 27, 18 shortlisted proteases had a percentage of cleavage events above 1% and are presented in Fig. 2a. Among the shortlisted proteases were multiple metalloproteinases (see Fig. 2a-in red), 4 proprotein convertases (see Fig. 2a-in blue), 2 cathepsins (see Fig. 2a-in green) and kallikrein 4 (KLK4). The 44% of the cleavage events were produced by 3 metalloproteinases (i.e. MMP13, MMP9 and MMP2). However, the most abundant diffrentially excreted peptides were putatively cleaved by the four proprotein convertases, KLK4 and MMP26, as shown in the heatmap (see Fig. 2b). For comparison, when analyzing the CRS-specific peptides (n = 193), similarly, 11 metalloproteinases and 4 cathepsins were predicted (see Fig. 2a marked with an asterisk), all included in the predictions from the 559 differentially excreted peptides.

Gene Ontology (Go) enrichment analysis of the 18 shortlisted proteases showed the over-representation of metalloproteinases (i.e. MMP3, MMP12, MMP14, MMP20, MMP26, MMP1, MMP2, MMP13, MMP9, MMP20, MEP1B, MMP8) in zinc ion binding and transition metal ion binding molecular functions. Together, metalloproteinases (i.e. MMP12, MMP13, MMP9) along with 2 cathepsins (i.e. CTSK, CTSS) displayed collagen-binding activity.

**Figure 3:**
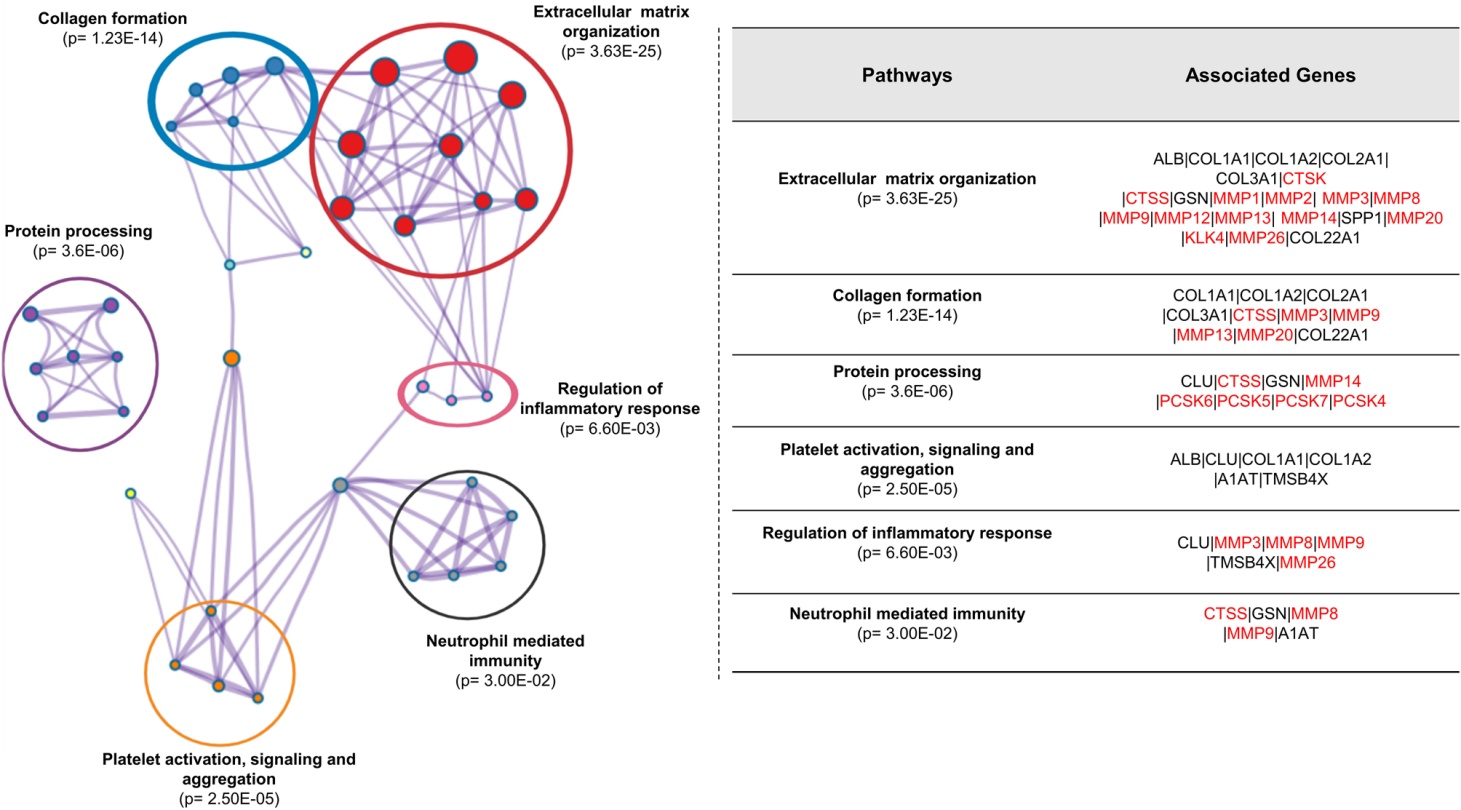
Pathway enrichment analysis. Pathway enrichment analysis of the 18 shortlisted proteases along with the 110 protein precursors of the differentially excreted peptides. The six most significant clusters are presented in different colours and the most significant term of each cluster was selected as label. For each term the corrected P-value along with the associated gene names are presented. The associated proteases are highlited in red colour.

Pathway enrichment analysis of the shortlisted proteases along with the protein precursors of the differentially excreted peptides was performed by Metascape. The analysis revealed that ECM-related pathways (i.e. ECM organization and degradation) were significantly affected. Together, cathepsins K and S along with various metalloproteinases (i.e. MMP1, MMP2, MMP3, MMP8, MMP9, MMP12, MMP13, MMP14, MMP20 and MMP26) and KLK4 were mostly involved in the structure, organization and degradation of ECM. Additionally, pathway analysis highlighted that the predicted proteases along with the protein precursors were also inolved in collagen formation, protein processing, regulation of inflammatory response and neutrophil mediated immunity (see Fig. 3).

## Discussion

In the present case–control matching study, a large-scale urine peptidome analysis of patients with CRS and matched controls was performed, targeting to identify the disease-specific urine peptidome profile and its potential links to pathophysiology. Importantly, our study identifies a high number of differentially excreted urine peptides between CRS patients and matched controls.

The results of the current study suggest that the urine peptidome of CRS patients integrates changes of 559 peptides originated from various protein precursors. These peptides originate to a large extent from proteins involved in ECM, collagen formation/degradation, inflammation, metabolism but also transcriptional regulation reflecting the underlying associated mechanisms^25^. A large number of overlapping urine peptides between CRS and HF as well as CRS and CKD patients were expected, as the examined patients of our study have combined both pathologies. These overlapping peptides derived, among others, from plasma proteins which are well-known renal and heart failure biomarkers including albumin, B2M, SPP1, AHSG, but also uromodulin and collagens^26^. In addition, to the above overlapping protein precursors, two proteins including, APOA1 (a protein which is associated with renal dysfunction in HF patients^27^) and FXYD2 (a protein which mediates the function of the Na, K-ATPase in mammalian kidney epithelial cells^28^) are commonly identified in CRS and CKD, similarly reflecting the common underlying molecular profiles of these diseases.

The highly abundant COL1A1, COL1A2, and COL3A1 proteins were represented by multiple urinary fragments, reflecting most likely fibrosis and changes in the ECM in both organs. In a healthy heart, the ECM is more than 95% composed of the fibrillar profibrotic collagens, including collagen types I and III^29^, important for maintaining the tensile strength, elasticity and extensibility of the myocardium^29^. Alterations of collagen type I and III expressions have been associated with myocardial fibrosis in HF patients^30^ and vascular calcification in the heart^31^ in animal studies. Similarly, collagen types I and III are the most abundant collagen types in the renal ECM^32^ linked to ECM dysregulation and CKD progression^33^. In addition, the activity of proteases may also be linked with the high number of the detected collagen-related peptides as it is well reported that metalloproteinases regulate cardiac and renal remodeling as well as fibrosis by facilitating ECM turnover and inflammatory cells^34,35^.

Further expected changes based on current knowledge were consistently detected. Among the most pronounced was the selective enrichment of increased albumin peptides in CRS, as expected for renal dysfunction and consequently albuminuria. Similarly, we observed an enrichment of peptides derived from COL9A3 and COL5A3, which are selectively highly expressed at the protein level in the heart and not detected in the kidneys based on proteomics databases (ProteomicsDB). Along the same lines, the abundance of a matrix GLA peptide (MGP) was strongly increased in CRS patients. MGP was already described as a calcification inhibitor protein with a strong association to HF indices and mortality^36^.

Similarly, in line with the literature, among protein precursors represented by decreased fragments in CRS patients were COL4A1 and COL4A3, collagens which are associated with nephropathy^37^. Furthermore, circulating proteins portrayed by increased fragments in CRS included AHSG (a protein which is associated with vascular calcification, cardiovascular mortality and kidney dysfunction^38^), APOA1 (a protein which is associated with HF and kidney dysfunction^27^), B2M (a protein which is associated with cardio-renal remodeling^11^ and inflammation^39^), COL18A1 (endostatin, generated from COL18A1, is associated with the development of cardiovascular events in CKD patients^40^), MGP and thymosin beta-4 (TMSB4X, a protein which is associated with renal fibrosis^41^).

Protein precursors detected only in CRS cohort but not in HF and CKD were also investigated. These included several collagen types, including COL4A2, COL4A4, COL6A5. Although few studies have aimed to investigate the role of collagen type IV and VI in cardio and renal failure, recently, it was suggested that COL4A2 is associated with cardiac fibrosis phenotype^42^, as well as with glomerular basement membrane alterations^43^, suggesting that COL4A2 may play an important role in CRS. In addition, COL4A4 and COL6A5 are associated with CKD^44,45^ but no evidence was found to link COL4A4 and COL6A5 with HF. Interestingly, two additional proteins, ROBO1 and HUWE1 are linked with cardiac and renal fibrosis^46,47^ whereas ANR17 protein may play a key role in the formation and maintenance of the blood vessels^48^. Moreover, several proteins associated with the immune system were uniquely found in the CRS cohort. These proteins were CD14 (a protein which is associated with heart and renal dysfunction^49^), CD99 and IRF6, suggesting the importance of the immune response in CRS. In addition, urinary PIGR peptides have been previously associated with cardiorenal dysfunction^50^, nevertheless, the exact role of the protein in the disease pathology remains unknown.

Our protease prediction analysis suggested the activity of 18 proteases responsible for more than 1% of the cleavage events. These predictions can be mostly supported by the existing bibliography on cardiovascular and renal pathologies. Notably, 9 out of the 11 predicted metalloproteases (MMP13, MMP9, MMP2, MMP20, MMP3, MMP12, MMP14, MMP8 and MMP1) were previously positively correlated with HF and were increased after myocardial infarction^34,51^. Interestingly, the transcriptional activation of MMP13 induces the vascular smooth muscle cell (VSMC) apoptosis and ECM breakdown via the FOXO3a activation^52^. These observations can be reflected by the contribution of these metalloproteases to vascular and kidney damage. Specifically, MMP2, MMP3, MMP8, MMP9 and MMP12 are involved in the ECM deposition in the glomeruli; MMP2, MMP3, MMP9, MMP13 and MMP14 induce epithelial-to-mesenchymal transition that leads to kidney fibrosis; and MMP2, MMP3 and MMP9 are correlated with vascular calcification, arterial stiffening and atherogenesis^53^. As reflected also by the cleavage events in our results, MMP2 and MMP9 have a prominent role in these processes and their levels have been associated with cardiovascular events and HF in various studies^54,55^. The role of MEP1B and MMP26 predicted by our analysis to mediate a large number of cleavage events, yet not previously linked to renal or heart dysfunction, apparently merits further investigation in the context of CRS.

From the 4 predicted proprotein convertases, three PCSK6 PSCK5 and PCSK7, have been previously associated with heart failure, to our knowledge. PCSK6 converts procorin to corin which in turn can activate natriuretic peptides, regulating cardiovascular and renal function^56^. Interestingly, PCSK6 is a key regulator of smooth muscle cell function (SMCs) in vascular remodeling and a novel player in cardiac remodeling after myocardial infraction^57,58^. Additionally, the inactivation of PCSK6 along with PCSK5 in endothelial cells leads to decreased collagen deposition and cardiovascular hypotrophy via IGF-1/Akt/mTOR signaling^59^. Moreover, PCSK7 is associated with both cardiovascular disease (CVD)^60^ and end-stage kidney disease^61^. However, the role of PCSK4 and KLK4 has not yet been linked to HF or CKD and would merit further investigation in the context of CRS based on our results.

Regarding cathepsins, elevated levels of CTSK were correlated with the presence of chronic HF^62^ and with major adverse cardiac and cerebrovascular events in CKD patients^63^, while circulating CTSS levels increase with CKD progression and GFR decline^64^. Levels of the mRNA, protein and activity of CTSS were found increased in the left ventricular myocardium of humans and rats with HF compared with controls, suggesting its participation in cardiac remodeling^65^. CTSS was also shown to affect epithelial-to-mesenchymal transition and ECM deposition in mouse models of mild and severe hydronephrosis, indicating its role in the regulation of renal fibrosis^66^.

Collectively, the pathology of CRS is complex and a number of pathways including ECM-related, fibrosis and inflammation are involved. Urinary peptidomics analysis reflects such CRS-associated changes, occasionally overlapping, as expected, with changes earlier observed in HF and CKD; but also alterations such as collagen type IV (COL4A2 and COL4A4), type VI (COL6A5), HUWE1, CD14, ANR17, PIGR and ROBO1 as well as a number of predicted proteases including MEP1B, MMP26, PCSK4 and KLK4, meriting further investigation in the context of CRS.

Among the strengths of our study was the large sample size allowing for meaningful patient matching. Based on this large sample size as well as on the case–control matching, we are confident in data validity and reliability. Additionally, all urinary peptides were analyzed using the same analytical platform and protocols (CE-MS). In contrast to any MS/MS approach, (CE-)MS does not provide sequence information. However, also as a result of the excellent reproducibility, the dataspace can be well defined, which results in the identification of over 4000 urine peptides and assignment of sequence based on mass and migration time with very high confidence^67^, which would not be possible based on LC–MS/MS. A detailed comparison of LC–MS/MS and CE-MS/MS is presented in Klein et al.^68^. In consequence, the approach combining highly reproducible CE-MS analysis with a large database on CE- and LC–MS/MS analyses, results in a much higher number of peptide identifications and in higher confidence in the results in comparison to LC–MS/MS only.

The study has some limitations: these include that it is retrospective, based on already available published data. However, the sample size, the multicenter design and the very high consistency and significance of the observed changes reduce the risk of this bias. Further, we limited our study to a subgroup of CRS: to subjects with CKD and HF. This is owed to the fact that we aimed towards depicting molecular changes. Such an approach requires homogeneity in molecular pathophysiology. In addition, a number of known kidney-specific and heart-specific protein biomarkers (such as KIM-1, L-FABP, NGAL, and NAG)^14^ could not be detected in our study; this is in fact expected as the applied technique (CE-MS) resolves the peptidome (< 10 kDa molecular mass peptides). Finally, the analysis is descriptive which, however, still opens multiple research avenues towards understanding the functional impact from the generation of the presented significant fragments.

In conclusion, this study reports the detection of a high number of urinary CRS-associated peptides when compared with controls. The underlying molecular mechanisms for the CRS pathology, as reflected by these peptides, represent fibrosis, ECM-related pathways, collagens formation/degradation and inflammation, in line with the existent knowledge. However, a number of peptides/protein precursors, not highlighted previously in association to HF or CKD, such as peptides of PIGR, CD14, ANR17, COL4A2 COL4A4, COL6A5, ROBO1 and HUWE1 may be important players in the mechanisms of CRS. As the present work is the first attempt to explore the urine peptidome profile of CRS patients, the findings of our study require further validation.

## Methods

### Study design: patient data and selection

Urinary peptidomics datasets from patients with CRS (in this study, CRS patients simultaneously combine both pathologies; HF and CKD), as well as individuals with no signs of diseases (controls) at urine sampling were used. These datasets corresponded to urine samples from cohorts described in several published studies investigating mainly renal failure (including Syskid^15^, FSGS-Aachen^24^), or heart and cardiovascular failure (including NTCVD-Urin^69^, FROG-ICU^70^, PCHF-Urin^71^ and HOMAGE^72^). The data were investigated for the availability of information on both kidney (i.e. CKD) and heart (i.e. HF) disease. The kidney function was assessed via the eGFR assessed based on ´Chronic Kidney Disease Epidemiology Collaboration´ (CKD-EPI). The ´European Society of Cardiology´ guidelines were used for the HF diagnosis and subtyping^73^. Clinical, pathophysiological, and molecular variables such as ejection fraction, systolic blood pressure, diastolic blood pressure, serum creatinine, hypertension, NT-proBNP levels, left ventricular ejection fraction (LVEF) as well as information on the age and sex, were retrieved (see Table 1). These datasets (corresponding to 3463 individuals) were then separated into the CRS cohort consisting of patients with both pathologies; HF and CKD with an eGFR < 60 ml/min/1.73 m^2^. The control group with no signs of heart disease and preserved kidney function (eGFR > 60 ml/min/1.73 m^2^) was selected (see Table 1). The study had received ethics approval (ΕΚ163/19 Ethik-Commission of the medical faculty of the RWTH Aachen), fulfilling all the requirements on the protection of the individuals participating in medical research and in accordance with the principles of the Declaration of Helsinki^74^. All data sets received were anonymized. All experiments were performed in accordance with relevant named guidelines and regulations. Each patient has written informed consent to use part of the tissue for scientific research.

### Case–control matching

CRS patients were matched on age and sex with controls at a 1:1 ratio. Following the case–control matching procedure, the final groups consisted of 353 patients with CRS compared with 356 individuals with no signs of either disease as listed in Table 1.

### CE-MS

The urine samples have been prepared and measured by CE-MS as stated before^75^. The P/ACE MDQ capillary electrophoresis system (Beckman Coulter, USA) connected to a micro-TOF–MS (Bruker Daltonic, Germany) was used for the CE-MS analysis. The probabilistic clustering algorithm along with isotopic distribution and conjugated masses for charge have been used for RAW MS data evaluation as described previously^67^. Totally, twenty-nine fragments of collagens that were not affected by disease were used for the normalization of the CE-MS data.

### Peptide sequencing

Urinary peptides were fragmented using Orbitrap MS coupled to CE (CE-MS/MS) or liquid chromatography (LC–MS/MS)^68^. The fragmentation spectra were matched to the protein sequences from up-to-date databases (International Protein Index^76^, Reference sequence database at NCBI^77^ and UniProt Knowledgebase^78^) using Proteome Discoverer 1.4 (Thermo Scientific, Bremen, Germany) with an integrated Sequest search engine^79^). The following search parameters were applied: (1) precursor mass tolerance: 5 ppm, (2) fragment mass tolerance: 50 mDa and (3) variable post-translational modifications: hydroxylation of lysine and proline, and oxidation of methionine, and (4) Enzyme name: No enzyme (Unspecific). Identified biomarkers were also assigned in silico to sequenced peptides from the Human Urinary Proteome database.

### Bioinformatic analysis

Information on the function and expression of the parental proteins was extracted from Gene Cards (http://www.genecards.org) and linked databases ProteomicsDB (http://www.proteomicsdb.org), Uniprot (http://www.uniprot.org), and Human Protein Atlas (http://www.proteinatlas.org). Pathway enrichment analysis and visualization of data were performed by the Metascape^80^. Gene Ontology (GO) annotation of molecular functions was performed by the Gene Ontology resource (GO; http://geneontology.org).

### Proteases analysis

The Proteasix (http://www.proteasix.org), an open-source tool was used for the protease prediction analysis^81^. As such, the potentially involved proteases were linked with the generation of the identified CRS-associated peptides. In brief, Proteasix uses information about the protease/cleavage site associations from a number of protease databases including the MEROPS, the CutDB, the UniProt Knowledgebase and the literature. The generated list of proteases is divided into two types; (a) “predicted” and (b) “observed”. For the “observed” proteases the protease/cleavage site association is collected from the literature, whereas for the “predicted” proteases the predicted proteolysis is determined by the MEROPS database. To improve the reliability of the proteolytic data, we decided to focus only on the “observed” proteases.

### Statistical analysis

The Kolmogorov–Smirnov normality test was used to determine the distribution of the urine peptidome data. Statistical analysis of the abundance of urinary peptides was performed using the non-parametric Mann–Whitney U test, followed by correction for multiple testing using the Benjamini-Hochberg (BH) method. Statistical analysis was performed using SPSS software version 20.0 (SPSS, Inc., Chicago, Illinois). A BH-adjusted P-value < 0.05 was considered to be statistically significant. The abundance of urinary peptides was analyzed and plotted using GraphPad Prism 7 (GraphPad Software, La Jolla, California, USA). Data are presented as mean ± standard deviation (s.d.) (*P < 0.05, **P < 0.01, ***P < 0.001, ****P < 0.0001).

## Supplementary Information

Supplementary Tables.

## Data Availability

All data generated or analysed during this study are included in this published article (and its Supplementary Information files).
